# Development of a plant-based vaccine against brucellosis: stable expression of *Brucella abortus* OMP25 in transgenic tobacco

**DOI:** 10.1007/s11248-025-00441-0

**Published:** 2025-04-29

**Authors:** Mansoure Qashqai, Emrah Bertan, Semiha Erisen, Tulin Ozbek, Senay Vural-Korkut

**Affiliations:** 1 Department of Molecular Biology and Genetics, Faculty of Arts and Sciences, Yıldız Technical University, 34220 Esenler, Istanbul, Turkey; 2 Institute of Science, Yıldız Technical University-Davutpasa Campus, 34220 Esenler, Istanbul, Turkey

**Keywords:** *Agrobacterium Tumefaciens*, Subunit vaccine, Gateway cloning, Transgenic plant

## Abstract

Brucellosis, caused by *Brucella* species, is a global threat to livestock farming, resulting in economic losses and socio-economic challenges, particularly in rural areas. Despite its impact, no licensed human vaccines are available. Animal vaccination remains the most cost-effective control method, but traditional vaccine production is expensive. Edible vaccines, using plants as bioreactors to produce immunogenic antigens, offer a low-cost alternative by eliminating complex purification processes. This study developed a transgenic plant by expressing the *Brucella abortus* outer membrane protein OMP25 in tobacco plants. OMP25, a conserved transmembrane protein with high immunogenicity, was cloned into a Gateway pDONR vector via a Boundary Pairing reaction and transferred to a binary destination vector via a Left–Right reaction. The destination vector was introduced into *Agrobacterium tumefaciens* and subsequently used for Agrobacterium-mediated transformation of tobacco plants. Transgenic plants were selected on media containing kanamycin, and the expression of the transgene was verified through the fluorescence of green fluorescent protein. Microcallus formation and shoot development on selective media confirmed kanamycin resistance and the successful integration of the transgene. After phenotypic selection, genomic DNA was extracted from transgenic plants and analyzed by PCR (Polymerase Chain Reaction) using primers specific to the *OMP25* gene. Positive PCR results validated the successful integration of the OMP25 gene into the plant genome. Gene expression was further confirmed at the RNA level through real-time quantitative PCR (qRT-PCR) and at the protein level via Western blot analysis. Future studies will evaluate immune responses in animal models. This approach demonstrates the potential for low-cost, effective vaccines to combat brucellosis, addressing critical economic and public health challenges.

**Supplementary Information:**

The online version contains supplementary material available at 10.1007/s11248-025-00441-0.

## Introduction

Brucellosis, caused by bacteria of the Brucella genus, is a globally significant zoonotic disease with profound implications for both veterinary and public health. In livestock, Brucella infection primarily induces reproductive disorders, including abortion, infertility, and decreased milk production, leading to substantial economic losses in the agricultural sector (Franc et al. 2018). The disease is particularly prevalent in developing countries, where insufficient surveillance, inadequate biosecurity measures, and limited access to vaccination programs contribute to its persistence as a neglected zoonosis (Pappas et al. 2006). The economic burden of brucellosis extends beyond direct losses in livestock productivity to include trade restrictions, increased veterinary costs, and potential transmission to humans, necessitating a comprehensive, interdisciplinary approach for effective control and eradication. he pathogenicity of Brucella is largely attributed to its intracellular lifestyle, enabling it to persist within host cells such as macrophages, dendritic cells, trophoblasts, also microglia, fibroblasts, epithelial, and endothelial cells. This mode of existence not only limits exposure to the host’s immune defenses but also shields the pathogen from the action of specific antibiotics, thus contributing to its unique virulence in infected hosts (Archambaud et al. 2010; Celli 2015). Following an asymptomatic incubation period, brucellosis progresses to an acute stage marked by widespread dissemination of the pathogen in host tissues, potentially evolving into a chronic phase that can result in severe organ damage and, in some cases, host death (Baud & Greub 2011). The treatment of brucellosis typically involves a combination of antibiotics; however, the emergence of antibiotic-resistant Brucella strains poses a significant challenge to effective disease management and control (Qureshi et al 2023). The pathogenicity of *Brucella* is largely attributed to its intracellular lifestyle, enabling it to persist within host cells such as macrophages, dendritic cells, trophoblasts, also microglia, fibroblasts, epithelial, and endothelial cells. This mode of existence not only limits exposure to the host’s immune defenses but also shields the pathogen from the action of specific antibiotics, thus contributing to its unique virulence in infected hosts (Archambaud et al. 2010; Celli 2015). Following an asymptomatic incubation period, brucellosis progresses to an acute stage marked by widespread dissemination of the pathogen in host tissues, potentially evolving into a chronic phase that can result in severe organ damage and, in some cases, host death (Baud & Greub 2011). The treatment of brucellosis typically involves a combination of antibiotics; however, the emergence of antibiotic-resistant *Brucella* strains poses a significant challenge to effective disease management and control (Qureshi et al. 2023).

Brucellosis persists with fluctuating morbidity and rising incidence, highlighting the need for a comprehensive surveillance strategy to control the epidemic. Effective brucellosis control strategies encompass a variety of approaches, including the culling of infected animals, mass vaccination, and monitoring of herds and agricultural lands for disease prevalence, as well as the establishment of brucellosis-free herds and regions. Among these, vaccination remains the most widely adopted and efficient method, particularly for cattle, globally. Over time, the advent of live attenuated strains has allowed for adaptation to changing epidemiological and socio-economic contexts, offering a more tailored approach to brucellosis control (Liu, et al. 2024; Corbel 2006).

Live attenuated vaccines are favored for combating intracellular pathogens such as *Brucella* spp. due to their ability to stimulate robust immune responses by promoting antigen production within the host. In Turkey, the *B. abortus* S19 vaccine, derived from the naturally attenuated S19 strain, plays a central role in brucellosis prevention strategies. While these vaccines effectively reduce virulence, they are not without limitations. Their relatively modest impact on preventing abortions in animals, emerging patterns of antibiotic resistance, cross-reactivity in serological tests leading to false positives, and the potential risk of transmission to humans all contribute to concerns regarding their pathogenicity. Furthermore, a significant challenge remains in distinguishing between immune responses induced by vaccination and those triggered by naturally acquired pathogenic strains (Cassataro et al. 2005; Heidary et al. 2022).

In the pursuit of safer and more effective vaccines, recombinant DNA technology has paved the way for the development of subunit vaccines, which offer a promising alternative to traditional vaccine approaches. However, these vaccines often require the implementation of diverse strategies to enhance immune responses, as they may induce suboptimal immunity on their own (Perkins et al. 2010). Compared to vaccines based solely on protein antigens, the inclusion of adjuvants in the formulation has been shown to provoke a stronger and more robust immune response (Velikovsky et al. 2002, 2003). This, in turn, may lead to the induction of more effective immune responses in vivo. These varied vaccine strategies underscore the presence of different immune mechanisms at play, highlighting the complexity and adaptability required to achieve optimal protection.

The selection of appropriate expression and vector systems is pivotal in the production of proteins intended for antigenic purposes. While prokaryotic expression systems, particularly those utilizing *Escherichia coli* strains, are traditionally employed due to their simplicity and cost-effectiveness, these systems face inherent limitations, such as the presence of endotoxins, protein folding that can lead to the formation of inclusion bodies limited secretion capacity, codon usage biases that can affect protein expression and restricted post-translational modification (PTMs) capabilities (Mitraki et al. 1991; Palomares et al. 2004; Kamoshida, et al., 2024; Yin et al. 2013). Over the past decade, plant-based expression systems have emerged as a compelling alternative, offering several advantages, including rapid production, low-cost scalability, enhanced post-translational modifications typical of eukaryotic systems, and the absence of contaminants such as mammalian pathogens. In plants, heterologous protein production can be achieved via stable or transient expression strategies. For our study, we prioritized transient expression systems, given their capacity to yield significant protein quantities within a short timeframe. These systems typically employ plant viral vectors, *Agrobacterium tumefaciens*, or a combination thereof, enabling the production of over 2 g of protein per kilogram of plant biomass within as little as one week. Such rapid and high-yield production capabilities have made plant-based systems particularly attractive for the development of antigens, notably those targeting COVID-19, as well as for therapeutic applications such as cancer treatment (e.g., monoclonal antibody BR-96 in soybean) and recombinant drugs/proteins like glucocerebrosidase (hGC) and interleukin-10 in tobacco. (Mahmood et al 2020; Jan et al. 2016). The burgeoning portfolio of biotechnologically relevant proteins produced in plants underscores their potential as a transformative platform in molecular pharming. These advancements pave the way for scalable, cost-effective, and safe solutions to address global challenges in healthcare and beyond.

This study aimed to produce the OMP25 protein, derived from the *OMP25* gene, in *Nicotiana tabacum* via *Agrobacterium tumefaciens*-mediated gene transfer for potential application in combating brucellosis. OMP25, an outer membrane protein highly conserved among Brucella species, was selected as the target antigen due to its critical role in host–pathogen interactions, a high antigenic index of 0.75, and its ability to exhibit optimal solubility and flexibility. These attributes collectively confer significant immunogenic potential to OMP25. Moreover, the structural flexibility of OMP25 enhances its immunogenic capacity by facilitating the recognition of multiple epitopes within the spatial configuration of immune cells. Previous studies have identified five B-cell and five T-cell epitopes within OMP25, highlighting its robust capacity to elicit strong immune responses. These findings have established OMP25 as a promising antigenic candidate for the development of vaccines against Brucella and underscored its pivotal role in inducing protective immunity (Gouran et al. 2023). Vaccination studies further demonstrate that OMP25 effectively enhances immune defenses and provides significant protection against *B. abortus* infections in murine models (Gouran et al. 2023; Akmayan et al. 2024). By leveraging the transient expression system in plants, this study seeks to address the critical need for scalable, cost-effective, and efficient antigen production platforms, ultimately contributing to advancements in vaccine development against brucellosis.

Recent advancements in Brucellosis vaccine development have underscored the critical role of OMP25 as a promising target for vaccine design. Gouran et al. (2023) demonstrated the use of *Lactococcus lactis* as a delivery platform to enhance OMP25 immunogenicity, while Rutkowska et al. (2024) identified *Nicotiana tabacum* as a viable and cost-effective system for the transient production of recombinant antigens.

In this study, we aimed to produce the OMP25 protein in *Nicotiana tabacum* using *Agrobacterium tumefaciens*-mediated gene transfer to address the critical need for scalable and cost-efficient antigen production. By achieving stable transformation, we successfully developed transgenic plants capable of continuously expressing OMP25, ensuring consistent and long-term antigen production for vaccine applications. This approach overcomes the inherent limitations of transient gene expression systems, providing a reliable and sustainable platform for large-scale vaccine production.

## Material and method

### Optimization and cloning of OMP25 sequence using the gateway system

The *OMP25* gene was retrieved from the NCBI database (https://www.ncbi.nlm.nih.gov/) and optimized for expression in *Nicotiana tabacum* using Genewiz (https://www.genewiz.com/). Gene transfer and expression were facilitated using the Gateway™ cloning system, which enables amplification of target DNA sequences by PCR with *attB*-specific primers to generate entry clones. LR recombination reactions subsequently created expression clones for protein production and functional analysis (https://www.molecularcloud.org/what-is-the-Gateway-Cloning.html).

The attB primers used for PCR amplification were:

Forward: 5′-GGGGACAAGTTTGTACAAAAAAGCAGGCTTCATGAGAACTCTTAAGTCTC-3′ Reverse: 5′-GGGGACCACTTTGTACAAGAAAGCTGGGTATTAAAACTTATATCCAATTCC-3′. The PCR product was purified, quantified, and recombined with the pDONR207 vector in a BP reaction. The resulting plasmids were transformed into chemically competent Escherichia coli cells using heat shock. Transformed cells were cultured on LB agar with 40 mg/mL gentamicin, and colonies were screened by PCR.

For LR recombination, the *pK7 WGF2* plasmid (Karimi et al 2002) was used as the destination vector. Reaction products were transformed into *E. coli*, cultured on spectinomycin-containing LB agar (125 mg/mL), and screened by PCR and plasmids were extracted from PCR-positive colonies.

### Transfer of the *OMP25* gene into *Nicotiana tabacum* plants by agrobacterium

The *OMP25* gene was introduced into *Agrobacterium tumefaciens* GV3101 via thermal shock transformation. Competent cells were mixed with the vector, subjected to liquid nitrogen freezing, and incubated at 37 °C. Transformed cells were cultured in LB medium, plated on selective LB agar (50 mg/L streptomycin (50 mg/mL) and spectinomycin (50 mg/mL), and incubated at 28 °C for 48 h. Colony PCR confirmed successful gene insertion and transformation.

The tobacco seeds used in this study were obtained from Aegean Agricultural Research Institute (Izmir/Turkey). The seeds were grown in pots including peat and perlite (3:1) watered with Hoagland’s solution (Hoagland & Arnon. 2007) in a controlled environment with a temperature of 25 ± 2 °C and light conditions of 16 h of light followed by 8 h of darkness, with an intensity of 90 mmol/m2/s, inside a growth room. Leaves from 4- to 6-week-old seedlings were sterilized by soaking them in a 20% commercial bleach for 30 min. and then three rinses with sterile distilled water for 5 min. Leaf disc placed in liquid MSD4 × 2 medium (which includes MSD supplemented with 0.1 mg/l NAA (Naphthalene Acetic Acid), 1 mg/l BAP (Benzyl aminopurine)) mixed with a1/50 dilution of A. tumefaciens culture (OD600: 0.8). After 30 min. explants removed on solid MSD4 × 2 medium at 25 °C for 2 days. After cocultivation they were cultured on regeneration medium (MSD4 × 2 including 50 mg/L kanamycin, 400 mg/L cefotaxime). Kanamycin-resistant microcallus appeared after two weeks, and shoots formed after four weeks. Shoots about 1 cm long were cut and moved to a rooting medium (MS medium supplemented with 0.2 mg/l NAA) with 50 mg/L kanamycin, 500 mg/L cefotaxime. Once roots formed, the plantlets were acclimatized to plastic pots containing 3:1 mixture of peat and perlite and kept in a growth chamber under the same controlled temperature and light conditions mentioned above.

### Assessment and verification of gene transfer efficiency

#### Phenotype selection and utilization of GFP as a reporter gene

Selection of transgenic plants was performed based on kanamycin resistance and GFP expression. Explants were cultured on a medium containing kanamycin (50 mg/L) to identify transgenic plants capable of growth under selective pressure, while wild-type explants were cultured under the same conditions to confirm the inhibitory effect of kanamycin on non-resistant plants. Additionally, both control and transgenic explants were cultured on MS agar medium without kanamycin to assess their growth under non-selective conditions. For further verification, shoots developing on selective medium were analyzed for GFP expression using a fluorescence imaging system. The presence of GFP fluorescence confirmed successful integration and expression of the transgene.

#### Molecular analysis of the candidate plants

Total DNA and RNA were extracted from transgenic and non-transgenic plants to confirm transgene integration and expression. DNA was isolated using the CTAB method, and PCR was performed to amplify a 135 bp region of the *OMP25* gene using specific primers (forward: 5′-CCTGCTCCTGTTGAAGTTGC-3′; reverse: 5′-AGCTCCAGCCTTCCAATCAT-3′). PCR conditions included initial denaturation at 94 °C for 2 min, 40 cycles of 94 °C for 20 s, 59 °C for 10 s, 72 °C for 40 s, and a final extension at 72 °C for 5 min. Products were analyzed on a 1.5% agarose gel at 100 V for 50 min and visualized under UV light to confirm transgene presence. RNA was extracted from leaf tissue using TRIzol Reagent (Thermo Fisher Scientific). The tissue was homogenized in liquid nitrogen and processed through chloroform extraction, isopropanol precipitation, and ethanol washing. RNA concentration and purity were assessed using a Nanodrop spectrophotometer. cDNA synthesis was performed using the SensiFAST™ RNA to cDNA Mix (BIO-65053), following the manufacturer’s protocol. Quantitative PCR (qPCR) was conducted with SYBR Green using β-actin as the reference gene (primers: forward 5’-CTGGCATTGCAGATCGTATGA-3’; reverse 5’-GCCACCTTGATCTTGGACAA-3’), and relative gene expression was calculated by the 2^-ΔΔCt method. All qPCR reactions adhered to MIQE guidelines, ensuring reproducibility and reliability. SDS-PAGE performed to assess the total protein expression, and Western blot analysis was conducted to detect GFP-OMP25 fusion protein expression in transgenic plants. Leaves from 7–8 leaf-stage transgenic and wild-type plants were collected and ground into a fine powder in liquid nitrogen for protein extraction. Two experimental groups were prepared by weighing 1 g and 2.5 g of leaf tissue, each supplemented with 3 mL of phosphate-buffered saline (PBS) containing Phenyl methyl sulfonyl fluoride (PMSF) as a proteinase inhibitor and incubated at 4 °C with gentle mixing for 90 min. Crude total soluble proteins (TSP) were obtained by centrifugation at 15,000 rpm for 20 min at 4 °C, and proteins with a molecular weight exceeding 10 kDa were isolated using ultrafiltration at 4 °C with a centrifugation speed of 4,000 g for 40 min. Protein concentrations were determined using the Easy I Protein Quantitative Kit (BCA assay). Approximately 20 µg of each protein sample were mixed with SDS loading buffer, heated at 95 °C for 5 min, and subjected to separation by 12% SDS-PAGE. The electrophoresis was performed at a constant voltage of 90 V for 1 h, followed by an additional hour at 125 V. The SDS-PAGE gel was subsequently stained with Coomassie Brilliant Blue to visualize the protein bands. The protein was subsequently electroblotted (30 min at 20 V). For immunoblotting, proteins were transferred to a 0.45 µm polyvinylidene difluoride (PVDF) membrane (Biorad, Cat. #1,620,167), blocked with 5% bovine serum albumin (BSA) for 1 h, probed with monoclonal anti-rabbit GFP antibody (1:500, Genetex, Cat. # GTX637570) and incubated with horseradish peroxidase-conjugated anti-rabbit IgG antibody (1:2,000, Biocompare, Cat. # ab207518), diluted in BSA. Membrane signals were visualized using the SuperSignal Chemiluminescent Substrate (ThermoScientific, Cat. # PI34578).

### Statistical analysis

Raw data analysis was performed using Microsoft Excel for initial data organization and management. GraphPad Prism 8.01 software was used for further statistical analysis. Differences between two groups were assessed using the ΔΔCt method for gene expression analysis. Gene expression data were evaluated with three biological replicates to ensure reliability and reproducibility, along with two technical replicates for each biological sample to account for technical variability. Normality of the data was assessed using the Shapiro–Wilk test. Since the prerequisites for parametric testing were not met, the Mann–Whitney U test was used as a non-parametric alternative. This methodological approach provided a rigorous framework for assessing gene expression levels. Statistical significance was determined at a threshold of p < 0.001, with differences considered statistically significant if the probability of observing such results by chance was less than 0.1%.

## Results

### Optimization, synthesis, and amplification of the OMP25 sequence for tobacco application

**Fig. 1:**
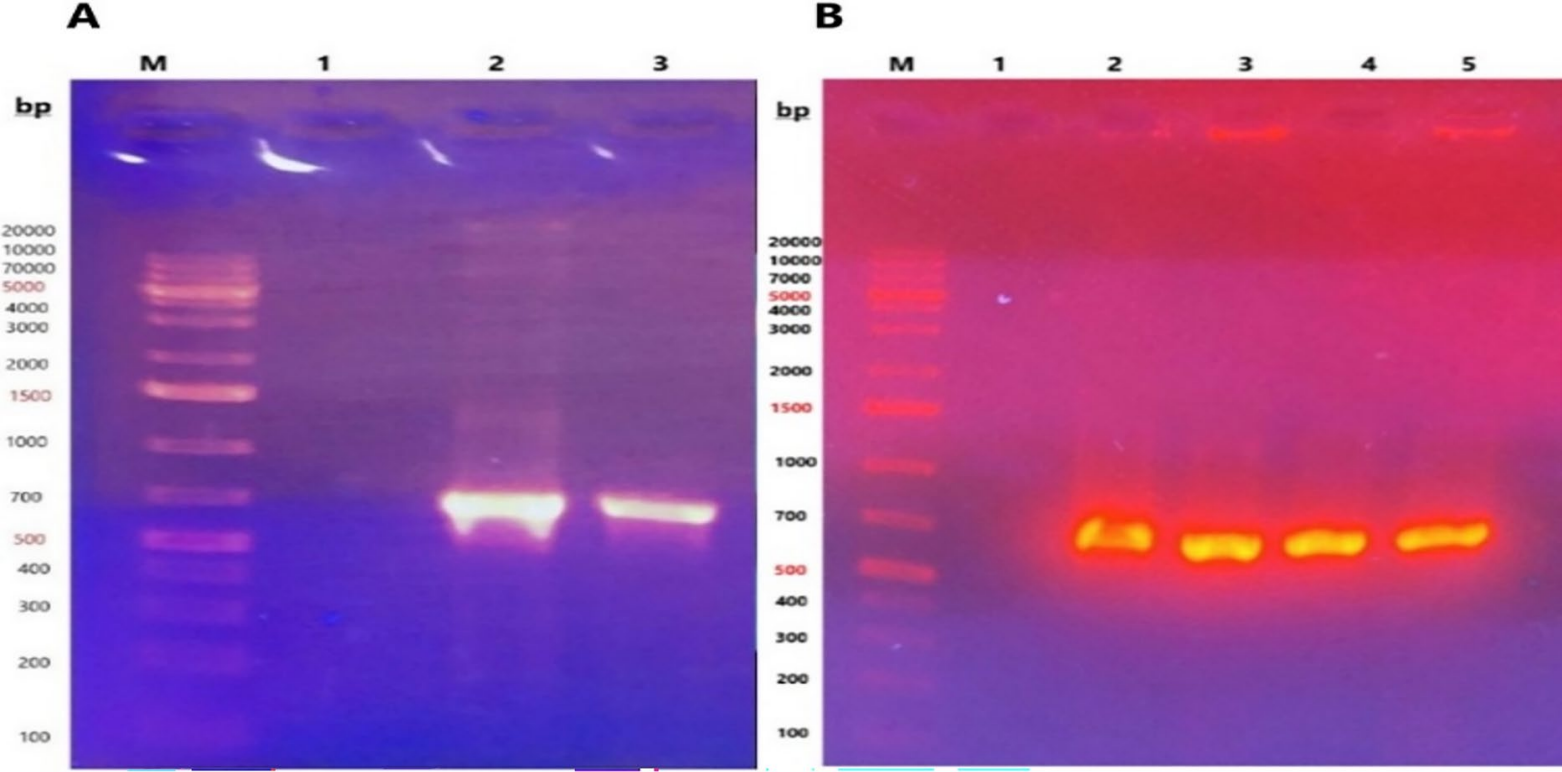
A. Amplified OMP25 by PCR: The image shows the amplicon size as determined using the GeneRuler DNA Marker. Lane M represents the DNA marker, Lane 1 contains negative control. Lanes 2 and 3 display the PCR products obtained from the experimental samples, was 743 base pairs (bp). **B**. Colony PCR: Four colonies were chosen for colony PCR analysis; Lane M represents the DNA marker. Lane 1 contains negative control. Lanes 2, 3, 4 and 5 display the PCR products obtained from the experimental samples, was 743 base pairs (bp) which confirmed that all selected colonies harbored the *OMP25* gene sequence

The present study successfully generated transgenic *Nicotiana tabacum* plants expressing the bacterialOMP25, a molecule implicated in enhancing immune recognition and response to infections. To optimize expression in the plant system, the *OMP25* gene sequence was codon-optimized for *Nicotiana tabacum*. The gene was amplified via polymerase chain reaction (PCR) using specific oligonucleotide primers. Agarose gel electrophoresis was employed to confirm the successful amplification of the *OMP25* gene, thereby validating the integrity of the synthetic construct (Fig. 1A). Molecular and biochemical analyses of the transgenic tobacco lines confirmed the expression of the OMP25 protein. This study underscores the feasibility of using plant-based systems for cost-effective and scalable protein expression.

### Construction of recombinant plasmid

The amplified *OMP25* gene was successfully ligated into the *pDONR207* vector and subsequently transformed into *Escherichia coli*. The transformed bacterial cells were cultured on LB agar plates supplemented with gentamicin and incubated at 37 °C for 21 h, resulting in the formation of bacterial colonies. To confirm the presence of the *OMP25* gene, colony PCR was performed on four randomly selected colonies. Electrophoretic analysis of the PCR products (Fig. 1B) demonstrated that all four colonies contained the *OMP25* gene sequence. One of the PCR-positive colonies was selected for further analysis and cultured in 20 mL of LB medium containing gentamicin to establish a working stock for subsequent applications.

After growing *E. coli* colony harboring the *pDONR207* vector with the OMP25 insert, the plasmid DNA was extracted using the BioLabs™ Plasmid Miniprep Kit. The extracted DNA yielded a concentration of 100 ng/µL with an A260/A280 ratio of 1.8, indicative of high purity. Sequencing of the PCR-amplified *OMP25* gene from the *pDONR207* plasmid confirmed 100% sequence similarity to the reference *OMP25* gene, with no observed alterations in the open reading frame or amino acid sequence that could impact the protein’s three-dimensional structure. The verified OMP25 sequence was subsequently cloned into the *pK7 WGF2* destination vector, which was transformed into E. coli. The transformed cells were cultured on LB agar plates containing spectinomycin and incubated at 37 °C for 21 h. Colony PCR analysis of three randomly selected colonies identified two positives for the *OMP25* gene. These positive colonies were cultured in LB medium with spectinomycin, and plasmid DNA was extracted, yielding a concentration of 45 ng/µL with an A260/A280 ratio of 1.8. The *pK7 WGF2* vector containing the OMP25 sequence was subsequently introduced into *Agrobacterium tumefaciens*. The transformed *A. tumefaciens* cells were plated on LB agar supplemented with spectinomycin and streptomycin and incubated at 28 °C for 48 h. Colony PCR confirmed the presence of the *OMP25* gene in all three tested colonies. These positive colonies were cultured in liquid LB medium containing spectinomycin and streptomycin and glycerol stocks were prepared and stored at − 80 °C for future use.

### Generation of transgenic *Nicotiana tabacum* plants

The leaf disc of *Nicotiana tabacum* plants (4–6 weeks old) were utilized for transformation. Leaf discs were sterilized and inoculated with *Agrobacterium tumefaciens* carrying the OMP25 construct. The discs were incubated on solid co- MSD4 × 2 medium at 25 °C for two days. Following this, the discs were transferred to a selection regeneration medium containing kanamycin, and cefotaxime. Under these conditions, kanamycin-resistant microcallus formed, and shoot regeneration was observed. Shoots reaching a length of 1–2 cm were excised and transferred to rooting medium supplemented with kanamycin, and cefotaxime. Root formation was initiated approximately one week after transfer. Out of 12 shoots placed on rooting medium, three successfully developed roots. Rooted plantlets were acclimatized by transferring them to pots and placing them in a greenhouse under control conditions. Of the three rooted plantlets, two exhibited successful adaptation, demonstrating clear signs of growth and development. These plants were deemed successfully established under growth chamber conditions.

### Verification of gene transfer of the *OMP25 *gene into *Nicotiana tabacum*

#### Phenotype selection and utilization of GFP as a reporter gene

Phenotypic selection of transgenic *Nicotiana tabacum* plants was performed based on kanamycin resistance and GFP fluorescence. The use of kanamycin resistance as a selectable marker ensured that only plants harboring the transgene survived and grew on media supplemented with the antibiotic. Additionally, GFP fluorescence provided visual confirmation of transgene expression. Under appropriate excitation conditions, the fluorescence indicated the successful integration and expression of the introduced genetic material in the selected plants. This dual-selection approach validated the presence and functionality of the transgene in the generated transgenic lines.

**Fig. 2:**
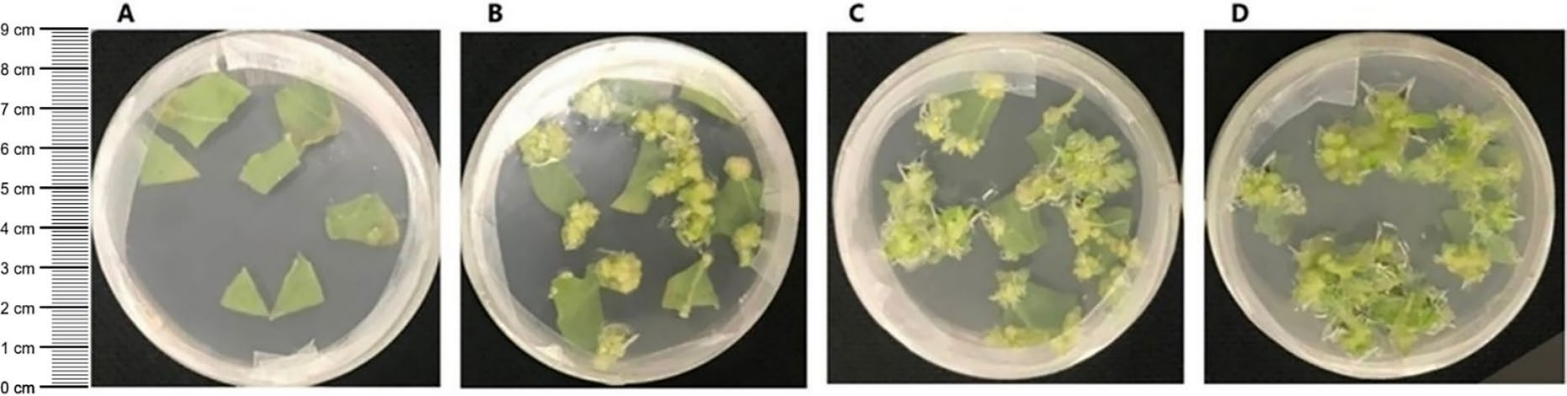
A. Control explants in kanamycin medium: Control explants showed no shooting, confirming kanamycin effectively inhibits the growth of non-resistant (wild type) plants. **Plate B**: Control explants in kanamycin- free medium: Control explants in kanamycin-free medium formed microcallus and shoots, indicating normal growth without selective pressure and their sensitivity to kanamycin. **Plate C**: Transgenic explants in kanamycin medium: Transgenic explants expressing the kanamycin resistance gene developed microcallus and shoots in kanamycin-containing medium, validating successful genetic transformation. **Plate D** Transgenic explants in kanamycin-free medium: Transgenic explants in kanamycin-free medium exhibited normal growth, consistent with their ability to grow in the absence of selective pressure

**Fig. 3:**
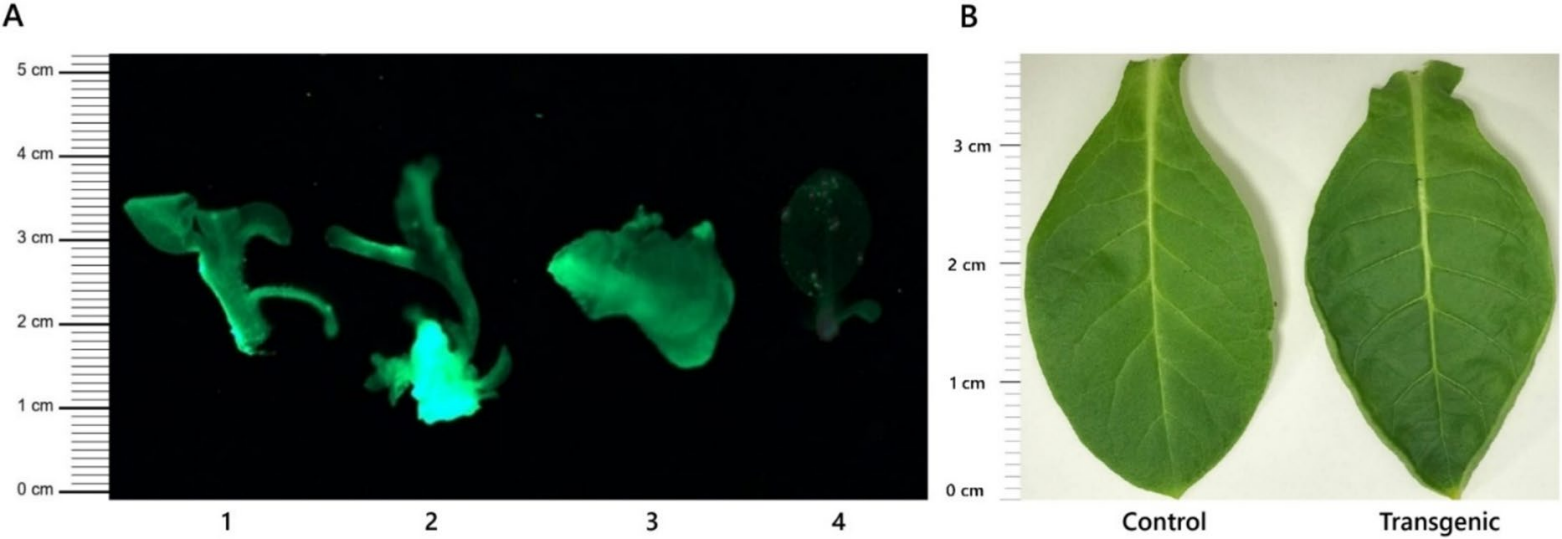
A. Fluorescence Analysis: Transgenic shoots (numbers 1, 2, and 3) fluoresced under UV(Ultraviolet) light due to GFP marker expression, directly confirming successful transgene expression. Wild type shoot (numbers 1) did not fluoresce, verifying the absence of transformation. **B**. Morphological Analysis: The transgenic plants showed significant leaf morphological changes compared to wild-type controls. The leaf shape shifted from the typical ovate form in wild-type plants to an obovate form in transgenic plants. Additional changes included zones with shallow depressions near the margins and inward curling of the leaf edges

To assess kanamycin tolerance, explants from putative transgenic and non-transgenic *Nicotiana tabacum* plants were cultured on MSD4 × 2 medium containing kanamycin and cefotaxime. The cultures monitored for eight weeks for regeneration. Microcallus formation was observed on explants from the transgenic plants within one week of culture initiation. After six weeks, shoot regeneration was evident from the microcallus, demonstrating the ability of the transgenic plants to grow and regenerate under selective conditions (Fig. 2C, 2D). In contrast, explants from wild-type control plants failed to form microcallus or regenerate shoots, confirming their inability to tolerate kanamycin (Fig. 2A). In contrast, microcallus and shoot formation are observed in control explants cultured in kanamycin-free medium (Fig. 2B). Additionally, GFP fluorescence was detected in the regenerated shoots from transgenic plants under fluorescence microscopy (Fig. 3.A). This observation provided direct evidence of successful transgene expression and further validated genetic transformation. The combined phenotypic selection and GFP fluorescence analysis confirmed the successful integration and functionality of the introduced transgene in the regenerated plants. Additionally, the transgenic plants exhibited several phenotypic changes, including leaf curling and an increase in leaf thickness. These alterations are likely a result of osmotic stress caused by the accumulation of the transgenic protein (Fig. 3.B).

#### Molecular analysis of the candidate plants

**Fig. 4:**
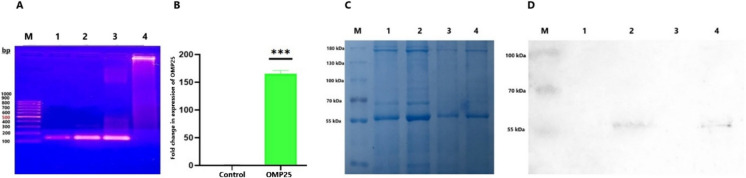
Verification of Transgene Integration and Expression in GFP-Expressing Transgenic Plants **A**. PCR Analysis: PCR was conducted to confirm the successful integration of the transgene into the genome of GFP-expressing transgenic plants by amplifying specific DNA sequences. The expected product size was 135 bp. Lane M represented the DNA marker used for estimating the size of the PCR products. Lanes 1 and 2 displayed PCR products of 135 bp from two transgenic plant candidates. Lane 3, the positive control, demonstrated successful amplification, while Lane 4, the negative control, showed no amplification. The presence of the *OMP25*gene sequence in both transgenic candidates was confirmed by the PCR results observed in Lanes 1 and 2. **B**. qPCR Analysis: To evaluate gene expression at the RNA level, Real-Time Quantitative Polymerase Chain Reaction (qPCR) was performed. Target gene expression, normalized to a reference gene, showed active transcription. Mean ± SEM (n = 3) indicated significantly higher OMP25 expression in the transgenic group vs. control (***p < 0.001). Error bars indicate the standard deviation. **C**. SDS-PAGE analysis: SDS analysis of recombinant GFP-OMP25 protein expression. Lane M, molecular weight marker; Lane 1 and 3, negative controls (wild-type plants), Lane 2 and 4, transgenic plant protein, with a band at 52 kDa corresponding to the GFP-OMP25 fusion protein. **D**. Western blot analysis: Western blot analysis of recombinant GFP-OMP25 protein expression. Lane M, molecular weight marker; Lane 1 and 3 negative control (wild-type plants), Lane 2 and 4, transgenic plant protein, with a band at 52 kDa corresponding to the GFP-OMP25 fusion protein

Genomic DNA was extracted from putative transgenic *Nicotiana tabacum* plants to confirm the integration of the *OMP25* gene. Polymerase Chain Reaction (PCR) was performed using primers specific to the *OMP25* gene. The amplification of the expected DNA fragment confirmed the successful integration of the *OMP25* gene into the plant genome (Fig. 4A). To assess transgene expression at the RNA level, Real-Time Quantitative PCR (qPCR) was conducted on RNA extracted from transgenic plant tissues. qPCR analysis detected OMP25-specific transcripts, indicating active transcription of the introduced gene. By normalizing the expression levels to a reference gene, the analysis confirmed that the transgene was being actively transcribed into RNA in the plant tissues (Fig. 4B). These results demonstrated that the *OMP25* gene was not only successfully integrated into the plant genome but was also transcriptionally active. This confirms the potential of the transgenic plants to produce the OMP25 protein, a critical step in evaluating their suitability as candidates for edible vaccines.

Protein extraction was performed on leaves from both transgenic and wild-type plants, with protein concentrations determined using the Easy I Protein Quantitative Kit (BCA assay). For the wild-type plants, protein concentrations were measured as 10.1 and 8.06 mg/mL for 1 g and 2.5 g leaf tissue samples, respectively, resulting in an average protein concentration of 7.94 mg/mL. In contrast, the transgenic plants exhibited protein concentrations of 8.93 and 11.5 mg/mL for the corresponding tissue weights, yielding an average protein concentration of 9.57 mg/mL.

To evaluate GFP-OMP25 expression, SDS-PAGE analysis was performed on 20 µg of protein from two biological replicates of transgenic plant samples and two negative controls. The desired band at approximately 52 kDa was observed in all samples, including both transgenic plant replicates and the negative controls (Fig. 4C). At the next step according to the SDS results, approximately 20 µg of each protein sample was subjected to SDS-PAGE analysis using a 12% polyacrylamide gel. Following electrophoresis, proteins were transferred to a polyvinylidene difluoride (PVDF) membrane and analyzed by Western blotting. The immunoblot revealed a distinct band at the expected molecular weight of 52 kDa, which corresponds to the recombinant GFP-OMP25 fusion protein. This band was clearly detectable in the transgenic plant samples, while no signal was observed in the wild-type samples, which served as a negative control (Fig. 0.4D). These results confirm the successful expression of the GFP-tagged OMP25 protein exclusively in the transgenic plants, validating the transgenic expression system.

These findings suggest that the transgenic plants can express functional protein and have the potential to serve as a viable system to produce plant-based vaccines. The successful expression of this bacterial protein highlights the capacity of these plants to function as an effective platform to produce a promising candidate for *Brucella* vaccination.

## Discussion

This study successfully developed a transgenic *Nicotiana tabacum* plant capable of expressing the OMP25 protein, a highly immunogenic component of *Brucella*. The production of OMP25 in plants represents a significant step toward the development of a plant-based subunit vaccine for brucellosis, the most widespread zoonotic disease globally. Brucellosis poses a severe threat to livestock health and productivity, while also representing a significant risk to human health in regions with close human-animal interactions. The plant-produced OMP25 subunit vaccine offers a promising and safer alternative to the currently employed live-attenuated *Brucella* vaccines. While these traditional vaccines have demonstrated efficacy, they are associated with several safety concerns, including the risk of pathogenic reversion in animals and potential zoonotic transmission to humans (Perkins et al. 2010).

Vaccination against *Brucella* infection has been extensively studied, with much of the focus on the use of live-attenuated strains for livestock. While these vaccines have provided a degree of protection, their limitations significantly impact their efficacy and practical application. Challenges associated with live-attenuated *Brucella* vaccines include interference with diagnostic assays due to the induction of LPS antibodies, residual virulence in humans, persistent infections in vaccinated animals, potential reversion to virulence, shedding in milk, and risks of abortion in pregnant animals even after a single dose. Moreover, these vaccines often fail to provide long-term immunity and protection, further limiting their effectiveness (Nicoletti 1990; Babaoglu et al. 2018). These drawbacks highlight the urgent need for the development of safer, more effective vaccines for brucellosis, with potential applications not only in livestock but also in humans. Subunit vaccines, which utilize highly immunogenic antigens such as OMP25, offer a promising alternative by addressing many of the safety and efficacy concerns associated with live-attenuated vaccines.

While significant advancements have been achieved in *Brucella* vaccine development, ongoing research continues to focus on overcoming the limitations of current vaccines. The development of plant-based subunit vaccines, as demonstrated in this study, represents an innovative approach to addressing these challenges. This strategy combines safety, scalability, and cost-effectiveness, offering a promising pathway toward the production of next-generation vaccines capable of addressing the shortcomings of traditional approaches. Recombinant vaccines aim to stimulate a targeted immune response using antigens, adjuvants, or viral/bacterial components, though inducing immunity against persistent infections remains challenging (Nascimento et al., 2012). Novel vaccines and antibodies against animal diseases produced in plants offer several advantages, including enhanced safety, proven efficacy, scalability, and relatively low capital investment. These characteristics make plant-based biopharming an affordable and accessible solution for developing countries (Murad et al. 2020; Topp et al. 2016; Tsekoa et al. 2020). Plant-based vaccine production is cost-effective, as it reduces purification costs and eliminates the risk of contamination by human pathogens. Additionally, vaccines derived from plants can be processed into low-cost powders or tablets, further improving their affordability and potential for widespread use (Su et al. 2023).

Currently, many plant-based vaccines are undergoing clinical trials for viral and bacterial pathogens. A vaccine incorporating Brucella melitensis T-cell epitopes onto Orbivirus core-like particles (CLPs), expressed in Nicotiana benthamiana, showed protective efficacy in a murine model (Rutkowska et al. 2024). However, a modified Brucella abortus RB51 strain failed to protect elk, highlighting the importance of selecting suitable antigenic proteins for effective vaccine development (Nol et al. 2016). These studies emphasize the need for careful selection of immunogenic components and the development of oral vaccines. OMP25 from Brucella species, identified as a promising candidate for vaccine development, induces both humoral and cellular immune responses. OMP25 was selected from 44 proteins for its solubility, structural flexibility, and antigenicity score (Gouran et al. 2023), showing strong potential in both innate and adaptive immune responses, particularly through IFN-γ production by Th1 cells. PLGA nanoparticles encapsulating recombinant OMP25 have demonstrated potential as an anti-Brucella vaccine (Akmayan et al. 2024).

The study by Atabey et al. (2021) explores immunogenicity of the recombinant OMP25 protein from Brucella abortus biovar 3, finding that it stimulates Th1-related cytokine release, highlighting its potential as a vaccine candidate for animal brucellosis. Given the scalability and biosafety of plant-based expression systems, we propose using stable expression of OMP25 in plants for oral vaccine development, offering a practical solution for Brucella prevention. We successfully developed transgenic tobacco expressing OMP25, addressing the challenge of transient gene expression. The observed phenotypic changes in transgenic plants, such as leaf curling and increased thickness, are likely a result of osmotic stress due to transgenic protein accumulation. This increased protein content may impose metabolic demands that influence cell wall structure, hormonal regulation, and growth patterns (Nieuwland et al. 2005; Wilson et al., 2016). Similarly, a study on EPO overexpression in transgenic tobacco and Arabidopsis reported retarded growth and male sterility, highlighting the physiological stress and metabolic burden caused by recombinant protein accumulation (Cheon et al; 2004). These phenotypic alterations are consistent with previous studies, which demonstrate that genetic transformation can significantly impact plant growth and development. Such modifications are likely driven by changes in cellular architecture, hormone signaling pathways, and metabolic fluxes (Fracasso et al. 2021). The correlation between enhanced protein expression and morphological changes suggests that transgene expression may not only influence protein accumulation but also have broader effects on overall plant physiology. Our approach offers several advantages over bacterial-based systems, including cost-effectiveness, simplicity, and the ability to trigger immune responses that allow differentiation between infected and vaccinated animals. Additionally, plant-based vaccines, especially edible ones, offer an easier delivery method, reducing the need for injections. Tobacco plants provide an environmentally friendly, scalable platform for recombinant protein production with minimal infrastructure and reduced contamination risks, making them a sustainable and accessible solution for brucellosis prevention, especially in resource-limited regions.

Future studies will look at the immune responses from these transgenic tobacco plants expressing the OMP25 protein in animal models. This approach could be a step toward creating a cost-effective, efficient, and safe vaccine for brucellosis. Using plant-based systems might help in the fight against brucellosis, a disease that affects both the economy and public health. These results could lead to new ways of addressing this ongoing challenge in animal and human health.

## Conclusion

In conclusion, this study is the first to develop transgenic tobacco plants expressing OMP25 as a potential edible vaccine. The expression of OMP25was confirmed using real-time PCR and Western blot analysis. Future research will involve animal model experiments to further evaluate the immune response triggered by these transgenic plants, helping to assess their potential as a vaccination strategy.

## Supplementary Information

Below is the link to the electronic supplementary material.

Supplementary file1 (DOCX 14 KB)

## Data Availability

The authors affirm that the data supporting the findings of this study are available within the article, and the sequencing results can be found in the supplementary materials.
