# Supplementary material for: Development of a plant-based vaccine against brucellosis: stable expression of Brucella abortus OMP25 in transgenic tobacco
Source: Transgenic Res. 2025 Apr 29;34(1):22. doi: 10.1007/s11248-025-00441-0 (PMC12041093; doi:10.1007/s11248-025-00441-0)
Supplement: Supplementary file 1 — Supplementary file1 (DOCX 14 KB) [file 11248_2025_441_MOESM1_ESM.docx]

>OMP25_F-OMP25

CTTGTCTGCTACTTCTTCCATTTTCTGCTACTGCTTTTGCTGCTGATGCTATTCAAGAACAACCACCTGTTCCTGCTCCTGTTGAAGTTGCTCCACAATATTCTTGGGCTGGTGGGTATACTGGACTTTATCTTGGATATGGATGGAATAAGGCTAAGACTTCTACTGTTGGATCTATTAAGCCTGATGATTGGAAGGCTGGAGCTTTTGCTGGATGGAATTTTCAACAAGATCAAATTGTTTATGGAGTTGAAGGAGATGCTGGATACTCTTGGGCAAAGAAATCTAAGGATGGACTTGAAGTTAAGCAAGGATTTGAAGGATCTCTTAGAGCTAGAGTTGGATATGATCTTAATCCTGTTATGCCATATCTTACTGCTGGAATTGCTGGATCTCAAATTAAGCTTAATAATGGACTTGATGATGAATCTAAGTTTAGAGTTGGATGGACTGCTGGAGCTGGACTTGAAGCGAAGCTTACTGATAATATTCTTGGAAGAGTTGAGTATAGGTATACGCAGTATGGAAATAAGAATTATGATCTTGCTGGAACTACTGTTAGAAATAAGCTTGATACTCAAGATATTAGAGTTGGAATTGGATATAAGTTTTAATACCCAGCTTTCTTGTACAAGTGGGTCCCCAAA

>OMP25_R-OMP25

ATCCAATTCCAACTCTAATATCTTGAGTATCAAGCTTATTTCTAACAGTAGTTCCAGCAAGATCATAATTCTTATTTCCATACTGCGTATACCTATACTCAACTCTTCCAAGAATATTATCAGTAAGCTTCGCTTCAAGTCCAGCTCCAGCAGTCCATCCAACTCTAAACTTAGATTCATCATCAAGTCCGTTATTAAGCTTAATTTGAGATCCAGCAATTCCAGCAGTAAGATATGGCATAACAGGATTAAGATCATATCCAACTCTAGCTCTAAGAGATCCTTCAAATCCTTGCTTAACTTCAAGTCCATCCTTAGATTTCTTTGCCCAAGAGTATCCAGCATCTCCTTCAACTCCATAAACAATTTGATCTTGTTGAAAATTCCATCCAGCAAAAGCTCCAGCCTTCCAATCATCAGGCTTAATAGATCCAACAGTAGAAGTCTTAGCCTTATTCCATCCATATCCAAGATAAAGTCCAGTATACCCACCAGCCCAAGAATATTGTGGAGCAACTTCAACAGGAGCAGGAACAGGTGGTTGTTCTTGAATAGCATCAGCAGCAAAAGCAGTAGCAGAAAATGGAAGAAGAGCAGCAGAAACAATAACAAGAGACTTAAGAGTTCTCATGAAGCCTGCTTTTTTGTACAAACTTGTCCCCACA
